# Endometrial compaction to predict pregnancy outcomes in patients undergoing assisted reproductive technologies: a systematic review and meta-analysis

**DOI:** 10.1093/hropen/hoae040

**Published:** 2024-06-20

**Authors:** Hannan Al-Lamee, Katie Stone, Simon G Powell, James Wyatt, Andrew J Drakeley, Dharani K Hapangama, Nicola Tempest

**Affiliations:** Department of Women’s and Children’s Health, Centre for Women’s Health Research, Institute of Life Course and Medical Sciences, University of Liverpool, Liverpool Health Partners, Liverpool, UK; Hewitt Centre for Reproductive Medicine, Liverpool Women’s NHS Foundation Trust, Liverpool, UK; Liverpool Women’s NHS Foundation Trust, Liverpool Health Partners, Liverpool, UK; Department of Women’s and Children’s Health, Centre for Women’s Health Research, Institute of Life Course and Medical Sciences, University of Liverpool, Liverpool Health Partners, Liverpool, UK; Department of Women’s and Children’s Health, Centre for Women’s Health Research, Institute of Life Course and Medical Sciences, University of Liverpool, Liverpool Health Partners, Liverpool, UK; Department of Women’s and Children’s Health, Centre for Women’s Health Research, Institute of Life Course and Medical Sciences, University of Liverpool, Liverpool Health Partners, Liverpool, UK; Department of Women’s and Children’s Health, Centre for Women’s Health Research, Institute of Life Course and Medical Sciences, University of Liverpool, Liverpool Health Partners, Liverpool, UK; Hewitt Centre for Reproductive Medicine, Liverpool Women’s NHS Foundation Trust, Liverpool, UK; Liverpool Women’s NHS Foundation Trust, Liverpool Health Partners, Liverpool, UK; Department of Women’s and Children’s Health, Centre for Women’s Health Research, Institute of Life Course and Medical Sciences, University of Liverpool, Liverpool Health Partners, Liverpool, UK; Liverpool Women’s NHS Foundation Trust, Liverpool Health Partners, Liverpool, UK; Department of Women’s and Children’s Health, Centre for Women’s Health Research, Institute of Life Course and Medical Sciences, University of Liverpool, Liverpool Health Partners, Liverpool, UK; Hewitt Centre for Reproductive Medicine, Liverpool Women’s NHS Foundation Trust, Liverpool, UK; Liverpool Women’s NHS Foundation Trust, Liverpool Health Partners, Liverpool, UK

**Keywords:** assisted reproductive technology, endometrium, endometrial compaction, endometrial thickness, endometrial receptivity, IVF, ICSI, pregnancy outcomes, progesterone, ultrasound

## Abstract

**STUDY QUESTION:**

Does endometrial compaction (EC) help predict pregnancy outcomes in those undergoing ART?

**SUMMARY ANSWER:**

EC is associated with a significantly higher clinical pregnancy rate (CPR) and ongoing pregnancy rate (OPR), but this does not translate to live birth rate (LBR).

**WHAT IS KNOWN ALREADY:**

EC describes the progesterone-induced decrease in endometrial thickness, which may be observed following the end of the proliferative phase, prior to embryo transfer. EC is proposed as a non-invasive tool to help predict pregnancy outcome in those undergoing ART, however, published data is conflicting.

**STUDY DESIGN, SIZE, DURATION:**

A literature search was carried out by two independent authors using PubMed, Cochrane Library, MEDLINE, Embase, Science Direct, Scopus, and Web of Science from inception of databases to May 2023. All peer-reviewed studies reporting EC and pregnancy outcomes in patients undergoing IVF/ICSI treatment were included.

**PARTICIPANTS/MATERIALS, SETTING, METHODS:**

The primary outcome is LBR. Secondary outcomes included other pregnancy metrics (positive pregnancy test (PPT), CPR, OPR, miscarriage rate (MR)) and rate of EC. Comparative meta-analyses comparing EC and no EC were conducted for each outcome using a random-effects model if *I*^2^ > 50%. The Mantel–Haenszel method was applied for pooling dichotomous data. Results are presented as odds ratios (OR) with 95% CI.

**MAIN RESULTS AND THE ROLE OF CHANCE:**

Out of 4030 screened articles, 21 cohort studies were included in the final analysis (n = 27 857). No significant difference was found between LBR in the EC versus the no EC group (OR 0.95; 95% CI 0.87–1.04). OPR was significantly higher within the EC group (OR 1.61; 95% CI 1.09–2.38), particularly when EC ≥ 15% compared to no EC (OR 3.52; 95% CI 2.36–5.23). CPR was inconsistently defined across the studies, affecting the findings. When defined as a viable intrauterine pregnancy <12 weeks, the EC group had significantly higher CPR than no EC (OR 1.83; 95% CI 1.15–2.92). No significant differences were found between EC and no EC for PPT (OR 1.54; 95% CI 0.97–2.45) or MR (OR 1.06; 95% CI 0.92–1.56). The pooled weighted incidence of EC across all studies was 32% (95% CI 26–38%).

**LIMITATIONS, REASONS FOR CAUTION:**

Heterogeneity due to differences between reported pregnancy outcomes, definition of EC, method of ultrasound, and cycle protocol may account for the lack of translation between CPR/OPR and LBR findings; thus, all pooled data should be viewed with an element of caution.

**WIDER IMPLICATIONS OF THE FINDINGS:**

In this dataset, the significantly higher CPR/OPR with EC does not translate to LBR. Although stratification of women according to EC cannot currently be recommended in clinical practice, a large and well-designed clinical trial to rigorously assess EC as a non-invasive predictor of a successful pregnancy is warranted. We urge for consistent outcome reporting to be mandated for ART trials so that data can be pooled, compared, and concluded on.

**STUDY FUNDING/COMPETING INTEREST(S):**

H.A. was supported by the Hewitt Fertility Centre. S.G.P. and J.W. were supported by the Liverpool University Hospital NHS Foundation Trust. D.K.H. was supported by a Wellbeing of Women project grant (RG2137) and MRC clinical research training fellowship (MR/V007238/1). N.T. was supported by the National Institute for Health and Care Research. D.K.H. had received honoraria for consultancy for Theramex and has received payment for presentations from Theramex and Gideon Richter. The remaining authors have no conflicts of interest to report.

**REGISTRATION NUMBER:**

PROSPERO CRD42022378464

WHAT DOES THIS MEAN FOR PATIENTS? Every percent counts when optimising fertility treatment and improving pregnancy rates for individuals and couples struggling to conceive. Endometrial compaction (EC) has been proposed as a novel, cheap, and non-invasive tool, using ultrasound technology, that could help with predicting pregnancy outcomes in patients undergoing some fertility treatments. EC refers to the decreased thickness of the womb lining, seen within the second half of the menstrual cycle of some individuals, in response to a rising hormone called progesterone. Progesterone rises in the second half of the menstrual cycle, around the time an embryo would usually implant. Conflicting data has been published regarding pregnancy outcomes associated with EC, with some studies reporting that EC is associated with significantly better pregnancy outcomes in those having fertility treatment and others showing no difference. By reviewing and analysing all of the available studies on EC and pregnancy outcomes in those having fertility treatment, we found that EC is associated with a significantly higher clinical pregnancy rate and ongoing pregnancy rate but is not significantly associated with an improved live birth rate, possibly due to the lack of available data. Therefore, although EC shows promise as an easily accessible and non-invasive tool that may be associated with improved pregnancy outcomes, due to the lack of translation to live birth rate, presently, EC should not yet be used as a prognostic aid in clinical practice. A future large clinical trial is required to robustly investigate the association between EC and pregnancy outcomes.

## Introduction

Increasing the live birth rate (LBR) of patients undergoing ART is the ultimate goal of any healthcare professional working within reproductive medicine. For successful implantation to take place, synchrony between both an embryo and a receptive endometrium is an essential requirement (Casper, 2020). The endometrium usually becomes receptive during the mid-secretory phase for a narrow period of time, commonly referred to as the ‘window of implantation’ (WOI) (Abdallah *et al.*, 2012). During this time, there is profound architectural remodelling, alongside transcriptional and secretional alterations within the endometrium, in response to the rise in circulating serum progesterone (Rosario *et al.*, 2003; Wang *et al.*, 2020).

Endometrial receptivity has been defined as a key factor in influencing IVF success, and an abnormal or displaced WOI has been proposed as a possible cause for recurrent implantation failure (RIF) and recurrent miscarriage (RM) (Casper, 2020). Pelvic ultrasound provides an accessible, cost-effective, and non-invasive method of assessing the endometrium (Bourne *et al.*, 1997). Endometrial assessment using ultrasound technology has therefore been proposed as a possible alternative method of predicting endometrial receptivity and successful pregnancy (Craciunas *et al.*, 2019).

First described by Haas *et al.* in 2019, studies now describe the occurrence of endometrial compaction (EC) in a subset of patients, referring to the progesterone-induced absolute decrease in endometrial thickness (EMT) seen between the end of the proliferative or oestrogenic phase and the day of embryo transfer (ET) (Haas *et al.*, 2019; Casper, 2020). As a new concept, little is known about EC, however, it is speculated to occur because of rising progesterone levels following ovulation, resulting in cessation of endometrial proliferation, and increased endometrial glandular development, immune cell proliferation, and angiogenesis, thereby increasing the density but not the volume of the endometrium (Fleischer *et al.*, 1984; Tabibzadeh, 1990; Bassil, 2001). Ultrasound follow-up of natural menstrual cycles shows that the EMT reaches a peak just before ovulation and then either plateaus or thins, giving rise to EC (Youngster *et al.*, 2022). Some studies looking at EC have attempted to correlate it with serum oestradiol and progesterone levels, however, poor correlation exists (Jarrah *et al.*, 2021; Olgan *et al.*, 2022; Youngster *et al.*, 2022; Ju *et al.*, 2023). Endometrial progesterone receptor deficiency, or resistance, may explain differences in EC among different patients/cycles as serum progesterone levels are not necessarily consistent with those within endometrial tissue (Usadi *et al.*, 2008; Lawrenz and Fatemi, 2022). In recent years, several studies have tried to determine if EC is linked to reproductive outcomes with inconsistent evidence published (Haas *et al.*, 2019; Zilberberg *et al.*, 2020; Riestenberg *et al.*, 2021; Yaprak *et al.*, 2021; Shah *et al.*, 2022). Some authors report no correlation between EC and pregnancy outcomes (Huang *et al.*, 2020, 2021; Jarrah *et al.*, 2021; Riestenberg *et al.*, 2021; Gursu *et al.*, 2022; Shah *et al.*, 2022), whilst others have observed a positive association between EC and pregnancy rates (Haas *et al.*, 2019; Zilberberg *et al.*, 2020; Kaye *et al.*, 2021; Yaprak *et al.*, 2021; Youngster *et al.*, 2022). If shown to be beneficial, EC could be a valuable way to help predict pregnancy outcomes in patients undergoing ART and could be an inexpensive method of guiding the timing of ET to synchronise with endometrial receptivity.

The primary aim of this study is to evaluate the association between EC and LBR. Within this systematic review and meta-analysis, we also aimed to robustly review and provide an up-to-date summary of the currently available evidence on the effect of EC on reproductive outcomes and the prevalence of EC within the sub-fertile population.

## Methods

This systematic review and meta-analysis was conducted in accordance with the Preferred Reporting Items for Systematic Reviews and Meta-Analysis (PRISMA) and Meta-analysis of Observational Studies in Epidemiology (MOOSE) guidelines (Stroup *et al.*, 2000; Page *et al.*, 2021). The protocol was prospectively written and registered with PROSPERO (registration number: CRD42022378464). The protocol did not require any revisions during the study period.

### Search strategy

A comprehensive literature search was conducted by two independent authors (H.A. and K.S.) for all studies published from inception to May 2023. PubMed, Ovid MEDLINE, Science Direct, Scopus, Embase, Web of Science, Cochrane Library, and Google Scholar databases were searched. The search strategy included the following Medical Subject Heading (MeSH) terms, keywords, and their combinations: ‘endometrium’ OR ‘endometrial’ AND ‘compaction’ OR ‘thickness’ AND ‘in vitro fertilisation’ OR ‘assisted reproductive techniques’. Additionally, database searches were supplemented with manual forward and backward citation chaining, and the ‘similar articles’ feature was searched on PubMed. Review articles were utilised to ensure all relevant citations were identified and included.

### Study selection and eligibility criteria

Duplicate articles were deleted, and the remaining articles were uploaded to Rayyan, an electronic review software (Ouzzani *et al.*, 2016) available from: https://www.rayyan.ai/. Two independent reviewers (H.A. and K.S.) performed title and abstract screening according to the pre-determined eligibility criteria. All original randomised and non-randomised studies assessing EC in association with any pregnancy outcomes in patients undergoing ART in the form of IVF/ICSI-ET cycles, including the transfer of either fresh or frozen embryos, were included in this review. Studies were excluded if: (i) they did not report on EMT or EC and any associated pregnancy outcome, (ii) they did not include study participants that had ART in the form of IVF/ICSI-ET cycles, (iii) were not written in the English language, (iv) were not full-text articles (including abstracts and incomplete datasets), and (v) were not original research studies (including review articles, meta-analyses, case-reports and conference abstracts). The remaining articles were subject to independent full-text review by the same two authors. In case of any disagreements, a third reviewer (N.T.) was consulted for resolution and discussed between all reviewers.

### Data extraction and synthesis

A standardised spreadsheet was developed and agreed upon between the authors. Selected studies were comprehensively examined, data extracted and recorded into the spreadsheet by H.A. and K.S. and then cross-checked by H.A. The data recorded included: author, year of publication, country of study, study aims, study design, sample size, experimental methods, outcome measures within the experimental group (EC) and comparator (no endometrial compaction (no EC)), study definitions of EC and outcomes parameters, and study conclusions. The primary outcome measure of this systematic review and meta-analysis was LBR. Secondary outcomes comprised of other pregnancy outcomes including positive pregnancy test (PPT), clinical pregnancy rate (CPR), ongoing pregnancy rate (OPR) miscarriage rate (MR), and endometrial compaction rate (ECR). Implantation rate (IR) and ectopic pregnancy rate (EPR) are not included as outcomes in this study due to a lack of studies reporting these outcomes. Definitions of pregnancy outcomes vary across the different studies. However, they are broadly defined as; PPT: either urinary or serum β-hCG detected at least five days following ET, IR: ratio of intrauterine gestation sac (IUGS) number over the number of embryos transferred, CPR: at least one IUGS detected with or without a foetal heartbeat (FH) at <12 weeks' gestation (where gestation was defined), OPR: viable pregnancy ≥12 weeks gestation, LBR: live birth of a fetus at least 22 weeks' gestation, MR: pregnancy loss <22 weeks' gestation (where gestation was defined), EPR: at least one gestation sac seen outside of the uterus. Further specific definitions of all pregnancy outcomes used within each study are outlined in Table 1. Currently, there is no universally accepted definition of EC; therefore, within this study overall, we defined EC as any decrease in EMT between the end of the oestrogen phase and the day of ET, allowing us to include all available study data on EC. Where possible, sub-analysis of different levels of EC and pregnancy outcomes was performed, using studies which reported on different degrees of EMT decrease/EC, to understand the impact on the results. Sub-group analysis of pregnancy outcomes at ≥5% EC, ≥10% EC, and ≥15% EC was performed. Corresponding authors were contacted where necessary if further clarity or data was required during the data extraction stage.

**Table 1:** Pregnancy outcomes measured by all studies, including study definition of outcome.

Study	Pregnancy outcome								
	Positive pregnancy	Implantation rate	Clinical pregnancy	Ongoing pregnancy	Live birth	Miscarriage	Ectopic pregnancy	Biochemical pregnancy	Preterm birth
Jarrah *et al.*, 2021	Serum β-hCG 14 days after ET (level >10 mIU/ml)	—	IUGS with FH <12 weeks’ gestation	Viable pregnancy ≥12 weeks’ gestation	—	Pregnancy loss <12 weeks’ gestation	—	—	—
Bu *et al.*, 2019	—	—	Not defined	—	—	Not defined	Not defined	—	—
Gill *et al.*, 2023	—	—	—	—	—	—	—	—	Delivery <37 weeks’ gestation
Gursu *et al.*, 2022	Serum β-hCG 5 days after ET (level >50 IU/ml)	—	IUGS with FH at 5 weeks after ET	—	LB of any fetus	Pregnancy loss <20/40	Any pregnancy outside of uterus	Falling β-hCG 48 h after first	—
Haas *et al.*, 2019	—	—	—	Not defined	—	—	—	—	—
Huang *et al.*, 2020	Serum β-hCG 14 days after ET (level ≥5 IU/l)	Ratio of IUGS number over the number of embryos transferred	At least one IUGS with or without FH at 7 weeks’ gestation	Viable pregnancy >12 weeks’ gestation	LB ≥24 weeks’ gestation	Pregnancy loss <12 weeks’ gestation	—	—	—
Huang *et al.*, 2021	Serum β-hCG 14 days after ET (level ≥5 IU/l)	Ratio of IUGS number over the number of embryos transferred	At least one IUGS with or without FH at 7 weeks’ gestation	Viable pregnancy ≥12 weeks’ gestation	LB >24 weeks’ gestation	Pregnancy loss after confirmation of clinical pregnancy <12 weeks’ gestation	—	—	—
Jin *et al.*, 2021a	—	—	Detection of one/more GS by US	—	At least one LB >22 weeks’ gestation	Pregnancy loss of an IUP <22 weeks’ gestation	—	—	—
Jin *et al.*, 2021b	—	—	Detection of one/more GS by US	—	At least one LB >22 weeks’ gestation	Spontaneous loss of IUP <22 weeks’ gestation	—	—	—
Ju *et al.*, 2023	—	—	One/more GS with a germinal bud and FH by US	—	—	—	—	—	—
Kaye *et al.*, 2021	—	—	—	US confirmation of FH at 12/40	—	Pregnancies that failed to achieve ongoing pregnancy	—	—	—
Lam *et al.*, 2022	Urine pregnancy test 16 days after ET	—	—	—	LB >24/40	Biochemical pregnancy and clinical miscarriage	—	—	—
Olgan *et al.*, 2022	Serum β-hCG 9 days after ET (level >10 IU/I)	—	One/more GS or trophoblastic tissue in a miscarriage specimen, or both	Pregnancy >12 weeks’ gestation	—	Spontaneous loss of IUP <12 weeks’ gestation, excluding those with biochemical pregnancy loss	—	—	—
Poojari *et al.*, 2023	Serum β-hCG 14 days after ET (without US evidence of pregnancy)	—	FH on US at 6 weeks’ gestation	—	—	—	—	—	—
Riestenberg *et al.*, 2021	—	—	FH on US	—	Not defined	Not defined	—	—	—
Shah *et al.*, 2022	—	—	At least one GS with a fetal pole and FH on TVS between 6 and 9 weeks’ gestation	—	Live birth ≥23 weeks’ gestation	—	—	—	—
Shaodi *et al.*, 2020	—	Number of clinical GS divided by total number of embryos transferred ×100%	At least one GS on US between 4 and 6 weeks after ET	—	One or more LB >28 weeks’ gestation	Miscarriage at <12 weeks’ gestation	Extra-uterine GS seen during US or surgery	—	—
Yaprak *et al.*, 2021	—	—	FH on US	—	—	—	—	—	—
Ye *et al.*, 2020	—	—	At least one GS in the uterine cavity at 4 weeks after FET	—	Delivery of infants >24 weeks’ gestation	Spontaneous pregnancy loss after visualisation of IUGS	At least one GS outside the uterine cavity by US	—	—
Youngster *et al.*, 2022	—	—	FH on US	FH on US >12 weeks’ gestation	—	—	—	—	—
Zilberberg *et al.*, 2020	—	—	—	FH on US ≥12 weeks’ gestation	—	—	—	—	—

ET, embryo transfer; FH, fetal heartbeat; FET, frozen embryo transfer; GS, gestation sac; IUGS, Intrauterine gestation sac; IUP, intrauterine pregnancy; LB, live birth; US, ultrasound; β-hCG, beta human chorionic gonadotrophin.

Results were statistically analysed with the aid of Review Manager (RevMan), Version 5.4, The Cochrane Collaboration, 2020. Relevant findings were summarised and discussed between all the authors allowing overall themes and conclusions to be drawn. Random effect models were used for meta-analyses unless the Higgins *I*^2^ statistic was <50%, indicating more homogenous datasets, in which case a fixed effect model was used. Meta-analyses are presented as forest plots. The Mantel–Haenszel method was applied for pooling of dichotomous data, and results were presented as odds ratio (OR) with 95% CIs (Mantel and Haenszel, 1959). An OR >1 for any outcome indicated a result in favour of the EC group and conversely, an OR <1 indicated a result in favour of no EC. Pooled weighted proportions of EC versus no EC across the studies were calculated using a random-effects model and the ‘metaprop’ command in Stata, version 14, StataCorp (Nyaga *et al.*, 2014). Summary proportions are presented with their corresponding 95% CIs. An overall effect *P*-value <0.05 was considered as statistically significant. Sensitivity analysis was performed by using the leave-one-out method for the primary outcome (LBR).

### Assessment of risk of bias

In order to assess the quality of all studies included, a risk of bias assessment was performed using the Newcastle-Ottawa scale (NOS) (Wells *et al.*, 2000). Each study was scored independently by two reviewers (H.A. and K.S.) between 0 and 9 stars based on three main areas: selection, comparability, and outcome, resulting in an overall quality assessment of ‘good’ (3–4 stars in the selection domain and 1–2 stars in comparability domain and 2–3 stars in the outcome domain), ‘fair’ (2 stars in the selection domain and 1–2 stars in comparability domain and 2–3 stars in the outcome domain), or ‘poor’ (0–1 stars in the selection domain or 0 stars in the comparability domain or 0–1 stars in the outcome domain). Any discrepancies were discussed and resolved by a third author (N.T.).

## Results

### Study selection

**Figure 1.**
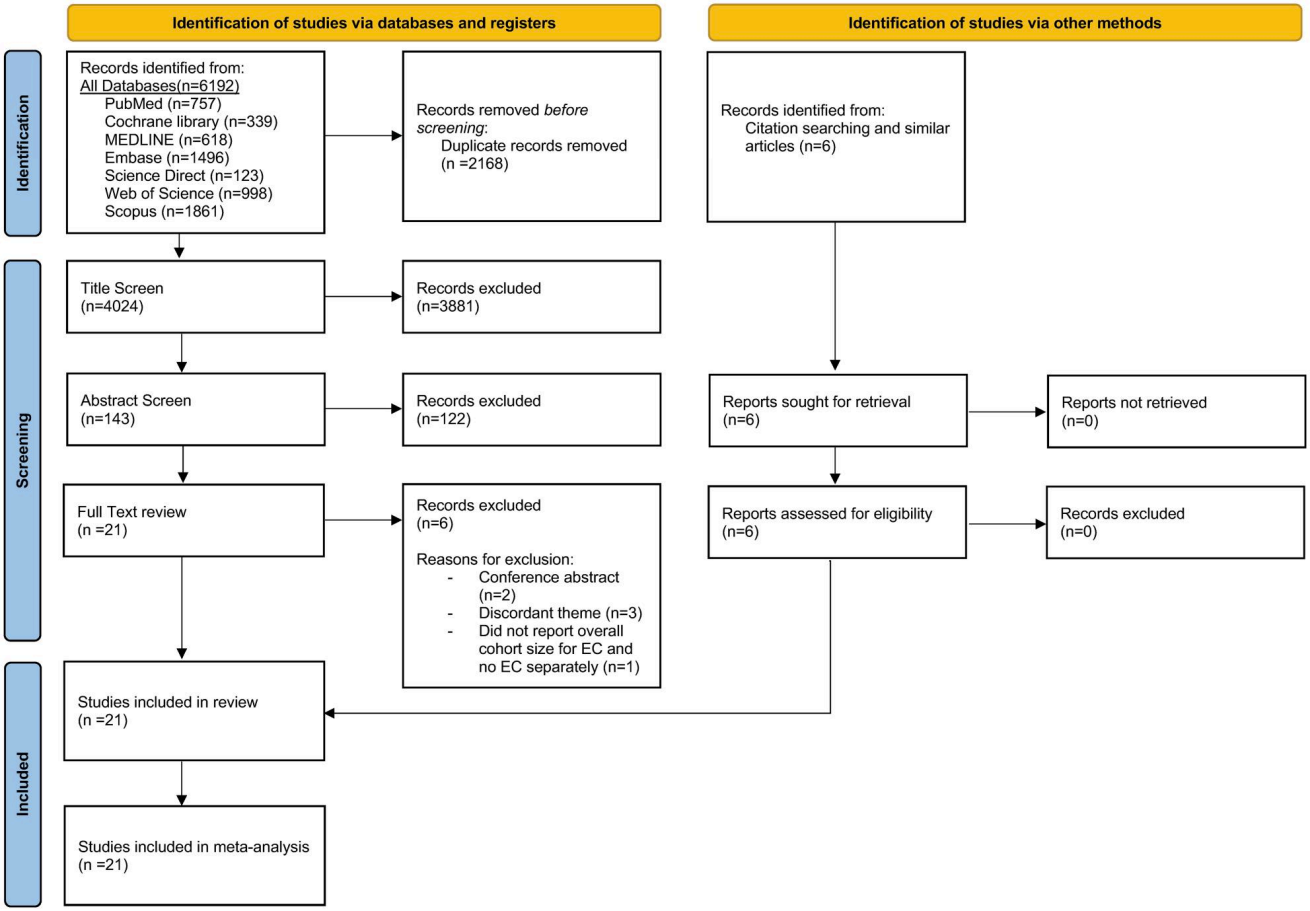
**PRISMA flowchart demonstrating the selection of publications identified in the systematic review and meta-analysis.** PRISMA, Preferred Reporting Items for Systematic Reviews and Meta-analyses.

A total of 6192 records were identified from the database searches and six by citation searching (Fig. 1, PRISMA diagram). Following the removal of duplicate records, 4024 records were eligible for title and abstract screening. An additional six studies were identified using forward and backward chaining. A total of 33 papers underwent full-text screening. Three studies were excluded as they had a discordant theme and two were excluded as they were conference abstracts only. One further study was excluded from the final analysis following data extraction, as they did not report an overall cohort size for EC and no EC, and we were unable to obtain this information when contacting the authors (Li *et al.*, 2022).

### Study characteristics

Twenty-one studies were included within the final analysis, with a total of 27 857 patients. The studies comprised of seven prospective and 14 retrospective observational studies, with no randomised controlled trials (RCTs) available on the topic. All studies were published over a 3-year period between 2019 and 2023. A summary of the study characteristics is provided in Table 2. Further details regarding pregnancy outcomes and study methods are provided in Tables 1 and 3.

**Table 2:** Summary of the study characteristics.

Reference	Location	Study period	Study design	Site	Treatment cycle type	Study n
Jarrah *et al.*, 2021	Iraq	2019–2020	Prospective cohort study	Single Centre	Medicated ICSI-FET cycles	60
Bu *et al.*, 2019	China	2015–2019	Prospective cohort study	Single Centre	Medicated and modified natural FET cycles	3091
Gill *et al.*, 2023	Canada	2016–2019	Retrospective cohort study	Single Centre	Fresh and FET ICSI cycles	252
Gursu *et al.*, 2022	North Cyprus	2017–2019	Retrospective cohort study	Single Centre	Fresh oocyte donation cycles	134
Haas *et al.*, 2019	Canada	2017–2018	Retrospective cohort study	Single Centre	Medicated FET cycles	271
Huang *et al.*, 2020	China	2011–2015	Retrospective cohort study	Single Centre	Modified natural FET cycles	2768
Huang *et al.*, 2021	China	2014–2019	Retrospective cohort study	Single Centre	Fresh IVF/ICSI cycles	2620
Jin *et al.*, 2021a	China	2014–2019	Retrospective cohort study	Single Centre	Modified natural FET cycles	219
Jin *et al.*, 2021b	China	2014–2019	Retrospective cohort study	Single Centre	Medicated ICSI-FET cycles	508
Ju *et al.*, 2023	China	2020–2022	Retrospective cohort study	Single Centre	Medicated FET cycles	1420
Kaye *et al.*, 2021	USA	2018–2019	Retrospective cohort study	Single Centre	Medicated FET cycles	232
Lam *et al.*, 2022	China	2005–2006	Retrospective cohort study	Single Centre	Fresh IVF/ICSI cycles	268
Olgan *et al.*, 2022	Turkey	2020–2021	Prospective cohort study	Single Centre	Medicated FET cycles	204
Poojari *et al.*, 2023	India	Not Specified	Prospective cohort study	Single Centre	Medicated FET cycles	156
Riestenberg *et al.*, 2021	USA	2018	Prospective cohort study	Single Centre	Medicated FET cycles	259
Shah *et al.*, 2022	USA	2020–2021	Prospective cohort study	Single Centre	Medicated and modified natural FET cycles	186
Shaodi *et al.*, 2020	China	2013–2017	Retrospective cohort study	Single Centre	Medicated FET cycles	10 165
Yaprak *et al.*, 2021	Turkey	2013–2019	Retrospective cohort study	Single Centre	Medicated FET cycles	283
Ye *et al.*, 2020	China	2010–2015	Retrospective cohort study	Single Centre	Medicated and modified natural FET cycles	4465
Youngster *et al.*, 2022	Israel	2019–2021	Prospective cohort study	Single Centre	Natural FET cycles	71
Zilberberg *et al.*, 2020	Canada	2016–2018	Retrospective cohort study	Single Centre	Medicated FET cycles	225

FET, frozen embryo transfer.

**Table 3:** Details of study design.

Study	Inclusion criteria	Exclusion criteria	Definition of EC	PGT	Study conclusion
Jarrah *et al.*, 2021	Age (20–40 years), BMI (19–35 kg/m^2^), normal uterine cavity/tubes, day 3 (grade A+/− B) embryos	RIF; pelvic pathology; EMT <7 mm; serum progesterone ≥1.5 ng/ml at end of follicular phase; follicular phase >21 days	Any decrease in EMT from end of follicular phase and day of ET	No	EC or serum progesterone levels measurements at ET were poor predictors for ongoing pregnancy. EMT changes seen after progesterone administration did not significantly affect pregnancy outcomes in FET cycles of cleavage stage embryos.
Bu *et al.*, 2019	Single blastocyst transfer, endometrial pattern A/B on the day of progesterone administration, and pattern C on the day of ET	Low quality embryo (blastocyst score <3 BC according to Gardner system); EMT <7 mm on day of progesterone administration; PGT cycles; OD cycles	Not defined	No	EMT on day of ET increased or was stable compared with that on day of progesterone administration in most patients. An increased EMT after progesterone administration was associated with better pregnancy outcome. Clinical pregnancy outcomes and the increasing rate EMT were positively correlated.
Gill *et al.*, 2023	Fresh ET/FET resulting in a single live birth >22 weeks’ gestation, images demonstrating longitudinal section of the endometrium and cervical canal	Gestational carriers; OD cycles; twin pregnancies; miscarriage/termination <22 weeks’ gestation; EMT <7 mm before starting progesterone; missing data	≥10% EMT decrease on the day of ET compared with EMT before starting progesterone	Not stated	Changes in EMT associated with EC during the WOI are not associated with an increased risk for placental or obstetric complications later in pregnancy.
Gursu *et al.*, 2022	Fresh OD cycles of recipients, normal semen analysis, screened egg donors (20–30 years) with no contraindication for donation	IVF cycles; FET/FET-OD cycles; consent declined; >2 embryos transferred; abnormal sperm parameters or any testicular interventions; EMT <7 mm despite 17 days of oestrogen or irregular endometrial pattern	Any decrease in EMT from day of initial progesterone administration to the day of ET	No	EMT change after 6 days of progesterone administration, whether increased or decreased, does not have any significant effect on LBR or CPR in fresh OD recipients.
Haas *et al.*, 2019	FET cycles	EMT <7 mm despite oestrogen treatment	≥10% decrease in EMT from 10 days after starting oestrogen and day of ET	Some (61 had PGT)	OPR showed a significant increase with each decreasing quartile of change in thickness (increased percentage EC) in the luteal phase compared with the follicular phase.
Huang *et al.*, 2020	Infertile women, regular ovulatory cycles, first FET cycle using modified natural FET protocol	After June 2015; history of RM; congenital uterine malformations; acquired uterine diseases; incomplete records; donor sperm; interval from egg collection to FET >1 year	>5% decrease from the day of hCG trigger to the day of ET	No	EMT change from hCG triggering to ET was not associated with pregnancy chances in modified natural FET cycles.
Huang *et al.*, 2021	First consecutive autologous IVF/ICSI cycles with fresh ET	History of spontaneous RM; congenital uterine anomalies; acquired uterine diseases; artificial oocyte activation; surgically retrieved spermatozoa; missing data	>10% decrease in EMT from day of hCG trigger to the day of ET	No	EMT change after hCG administration did not provide significant prognostic information for pregnancy outcome in fresh IVF/ICSI cycles.
Jin *et al.*, 2021a	Women with chromosome translocation/monogenic disease while the men were normal, PGT, first single FET of euploid blastocyst	Routine hysteroscopy showing the presence of uterine pathology; endometriosis; EMT< 7 mm on the day of progesterone administration; missing endometrial data	Not defined	Yes	In modified natural euploid blastocyst FET cycles the CPR was higher in cycles without EC after progesterone administration.
Jin *et al.*, 2021b	Women who received PGT for chromosomal structural rearrangements or PGT for monogenic/single gene defects and conducted their first single HRT FET	GnRHa used in advance for down-regulation for thin EMT in controlled ovarian stimulation cycles, hysteroscopy showing uterine pathology, endometriosis, EMT <7 mm on progesterone administration day, missing data	≥5% decrease in EMT	Yes	In medicated euploid blastocyst FET cycles the EMT change ratio after progesterone administration was not related to pregnancy outcomes.
Ju *et al.*, 2023	Age (20–49 years), artificial FET cycle	Concurrent systemic diseases; chromosomal abnormalities in either partner; endometriosis; congenital uterine malformations; untreated endometrial lesion; OD cycles; PGT	≥5% decrease in EMT	No	CPR was significantly higher in women with EC on ET day compared to women with no changes or increase in EMT.
Kaye *et al*., 2021	Autologous transfer of a single thawed blastocyst in medicated FET cycle	Failure to meet the inclusion criteria	≥10% decrease in EMT	Some (116 had PGT)	The change in EMT after progesterone initiation was associated with the probability of ongoing pregnancy but not with early pregnancy loss. OPR was greater in those with EC when compared to those with increasing EMT after progesterone exposure.
Lam *et al.*, 2022	Infertile women undergoing the first IVF/ICSI cycle with fresh ET	Abnormal uterine cavity; no ultrasound measurement of EMT and endometrial volume on both hCG and ET day	Not defined	No	EMT and EC was not a significant predictor of live birth in the multivariate logistic regression model. EMT and EC did not affect the LBR in fresh IVF cycles.
Olgan *et al.*, 2022	Medicated FET cycles using high-quality blastocysts for transfer	Medicated FET cycles with PGT; each patient included in the study once; thin EMT; endometrial polyp; endometrial fluid; misuse of drugs; COVID-19 diagnosis; post-thaw non-viable embryo; social reasons; medical problems	≥5% decrease in EMT. Expansion defined as a ≥ 5% increase. Cycles in which the percentage change was less than 5% were classified as no change cycles.	No	EC during medicated FET cycles does not predict ongoing pregnancy.
Poojari *et al.*, 2023	Age (<40 years), normal baseline uterine US/endometrial cavity, good quality day 3 embryo for transfer, EMT on day 14 of 7 mm on TVS	History of >2 miscarriages; two failed ETs; uterine fibroids; severe adenomyosis; septate uterus; history of Asherman’s syndrome; endometrial polyp	≥5% decrease in EMT	No	A significant increase in CPR was observed in women who had EC with good vascularity of the endometrium, unlike those with poor vascularity. Women with no change or increase in EMT had poor pregnancy rates.
Riestenberg *et al.*, 2021	ICSI-FET cycles, first ET per patient	Natural cycle and minimal-stimulation FETs; EMT <7 mm at the end of the follicular phase prior to initiation of progesterone	≥5% disease in EMT. Expansion defined as a ≥ 5% increase. If percent EC < ±5% it was considered unchanged	Yes	Most cycles did not demonstrate EC. EC is not associated with LBR or spontaneous MR in medicated single euploid FETs cycles.
Shah *et al.*, 2022	Age (18–43 years), first or second autologous single euploid FET	EMT <7 mm; BMI (>40 or <18.5 kg/m^2^); gestational carrier; history of ≥ 2 spontaneous abortions; current or prior uterine factors; those with prior endometrial receptivity analysis biopsy	Any decrease in EMT	Yes	LBRs were similar in participants who demonstrated EC or no EC.
Shaodi *et al.*, 2020	Medicated FET protocol, at least one good quality embryo transferred during each cycle	Chromosomal abnormality in either partner; uterine malformation; intrauterine conditions (endometrial polyps/uterine adhesions/history of endometrial tuberculosis/hydrosalpinx with a reflux into cavity); PGT; cycles of spontaneous ovulation	Not defined	Not stated	In medicated FET cycles, the optimal LBR would be obtained when the EMT remains within the range of 8.7–14.5 mm. If the endometrium is too thin or too thick, the LBR will be reduced.
Yaprak *et al.*, 2021	Artificial endometrial preparation, age (20–38 years), transfer of good quality embryos	Transfer of low-quality embryos; EMT <7 mm on the day of progesterone administration	Any decrease in EMT	No	EC is associated with a significantly higher LBR. EMT >9.2 mm at the end of the follicular phase is associated with a significantly increased chance of EC.
Ye *et al.*, 2020	Age (≤40 years), BMI (<30 kg/m^2^), first FET cycle, day 3 ET	Congenital/acquired uterine malformation; endometriosis; adenomyosis; endometrial polyps/submucosal fibroids	Not defined	Not stated	No significant difference in CPR and LBR regardless of EMT increasing, decreasing or remaining stable after progesterone administration. CPR and LBR did not undergo significant changes with the increasing ratio of EMT change regardless of medicated or natural cycles.
Youngster *et al.*, 2022	Age (≤40 years), natural FET cycle	Irregular cycles/cycles <21 or >35 days; BMI (>35 kg/m^2^); RIF/RM; poor ovarian response (Bologna criteria); severe endometriosis, tubal factor; uterine factor; PGT-A/PGT-M; OD; >2 prior ETs since last pregnancy; EMT on day 0/−1 < 7 mm or not trilaminar or endometrial fluid on the day of ET; vaginal bleeding prior to ET	>5% decrease in EMT	No	Around half of the patients in the study undergoing natural cycle FET cycles experienced EC. This was significantly correlated with increased CPR and OPR.
Zilberberg *et al.*, 2020	Not specified	EMT <7 mm after the follicular phase; images without the cervical canal; inaccurate EMT	≥15% decrease in EMT	Yes	EC in FET cycles with a single euploid ET resulted in a significant increase in OPR compared with cycles with no EC.

CPR, clinical pregnancy rate; EC, endometrial compaction; EMT, endometrial thickness; ET, embryo transfer; FET, frozen embryo transfer; GnRHa, gonadotropin-releasing hormone agonist; LBR, live birth rate; OD, oocyte donor; OPR, ongoing pregnancy rate; PGT-A, pre-implantation genetic testing for aneuploidy; PGT-M, pre-implantation genetic testing for monogenic disorders; PGT, pre-implantation genetic testing; RIF, recurrent implantation failure; RM, recurrent miscarriage; TVS, transvaginal ultrasound; US, ultrasound.

### Quality assessment and risk of bias

The NOS was used to perform a quality assessment on all studies included in this meta-analysis (Table 4). All studies were deemed to be of good quality following assessment, indicating reliable data.

**Table 4:** Risk-of-bias assessment for cohort studies using the Newcastle-Ottawa scale (NOS).

Author, year	Selection				Comparability		Outcome			Quality assessment	
	1	2	3	4	5	6	7	8	9	Total	Quality
Jarrah *et al.*, 2021	*	*	*	*	*	*	*	*	*	9	Good
Bu *et al.*, 2019	*	*	*	*	*	*	*	*	*	9	Good
Gill *et al.*, 2023	*	*	*	*	*	*	*	*		8	Good
Gursu *et al.*, 2022		*	*	*		*	*	*		7	Good
Haas *et al.*, 2019	*	*	*	*	*	*	*	*	*	9	Good
Huang *et al.*, 2020	*	*	*	*	*	*	*	*		8	Good
Huang *et al.*, 2021	*	*	*	*		*	*	*		7	Good
Jin *et al.*, 2021a	*	*	*	*		*	*	*	*	8	Good
Jin *et al.*, 2021b	*	*	*	*	*	*	*	*	*	9	Good
Ju *et al.*, 2023	*	*	*	*	*	*	*	*	*	9	Good
Kaye *et al.*, 2021	*	*	*	*	*		*	*	*	8	Good
Lam *et al.*, 2022	*	*	*	*		*	*	*	*	8	Good
Olgan *et al.*, 2022	*	*	*	*	*	*	*	*	*	9	Good
Poojari *et al.*, 2023	*	*	*	*	*	*	*	*	*	9	Good
Riestenberg *et al.*, 2021	*	*	*	*	*	*	*	*	*	9	Good
Shah *et al.*, 2022	*	*	*	*	*	*	*	*	*	9	Good
Shaodi *et al.*, 2020	*	*	*	*	*	*	*	*		8	Good
Yaprak *et al.*, 2021	*	*	*	*	*		*	*	*	8	Good
Ye *et al.*, 2020	*	*	*	*	*	*	*	*		8	Good
Youngster *et al.*, 2022		*	*	*	*		*	*	*	7	Good
Zilberberg *et al.*, 2020	*	*	*	*	*		*	*	*	8	Good

Risk-of-bias assessment for cohort studies using the NOS. Selection: (1) representative of exposed cohort, (2) selection of non-exposed cohort, (3) ascertainment of exposure, (4) demonstration that outcome of interest was not present at the start of the study. Comparability: (5) study controls for age, (6) study controls for any additional factors. Outcome: (7) assessment of outcome (8) was follow-up long enough for outcomes to occur (9) adequacy of follow-up of cohorts.

### Reproductive outcomes

#### Live birth rate

**Figure 2.**
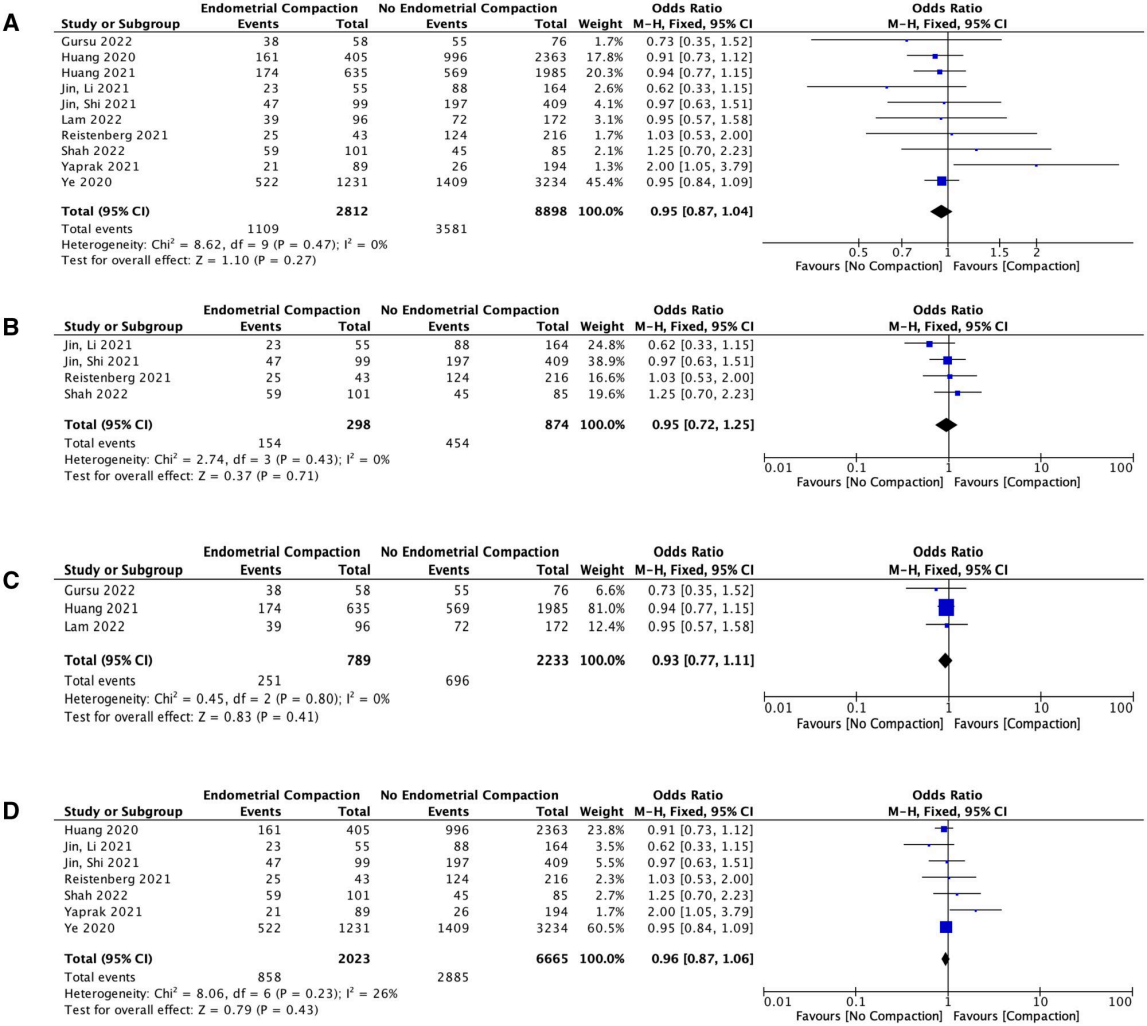
**Forest plots to show meta-analysis of LBR between EC versus no EC groups.** (**A**) LBR between EC versus no EC. (**B**) LBR in PGT studies only. (**C**) LBR in fresh cycles only. (**D**) LBR in FET cycles only. LBR, live birth rate; EC, endometrial compaction; PGT, pre-implantation genetic testing; FET, frozen embryo transfer.

Ten studies reported on LBR (2812 in EC group; 8898 in no EC group), showing no significant difference in LBR between the two groups (OR 0.95; 95% CI 0.87–1.04; *P *=* *0.27) (Fig. 2A). When sub-analysing this data based on level of EC, this also showed no significant difference in LBR between ≥5% EC and no EC (OR 0.58; 95% CI 0.29–1.19; *P *=* *0.14), ≥10% EC and no EC (OR 0.60; 95% CI 0.35–1.02; *P *=* *0.06) or ≥15% EC and no EC (OR 0.40; 95% CI 0.07–2.15; *P *=* *0.29). The same result was observed when sub-analysing the four studies that included euploid embryos only (OR 0.95; 95% CI 0.72–1.25; *P *=* *0.71) (Fig. 2B). Sub-analysis of fresh ET (three studies) versus FET cycles (seven studies) also showed no significant differences in LBR regardless of ET protocol (fresh ET cycles: OR 0.93; 95% CI 0.77–1.11; *P *=* *0.41 (Fig. 2C); FET cycles: OR 1.12; 95% CI 0.78–1.62; *P *=* *0.54) (Fig. 2D). Further sub-analysis of FET cycles only, based on whether they were natural cycle (NC) FET or HRT FET cycles, also showed no significant differences in LBR between EC and no EC for either cycle type (NC FET cycles: OR 0.90; 95% CI 0.74–1.10; *P *=* *0.31; HRT FET cycles: OR 0.98; 95% CI 0.87–1.11; *P *=* *0.75). Sensitivity analysis using the leave-one-out approach presented similar results, with no single paper found to alter the results significantly.

#### Positive pregnancy test

Seven of the included studies reported on PPT (631 in EC group; 2540 in no EC group). No significant difference was demonstrated between EC and no EC groups (OR 1.54; 95% CI 0.97–2.45; *P *=* *0.07) (Supplementary Fig. S1A). Sub-analysis of FET cycles only included five studies (324 in EC group; 1617 in no EC group) which showed no significant difference between the groups (OR 2.27; 95% CI 0.78–6.61; *P *=* *0.13) (Supplementary Fig. S1B). Four studies were further sub-analysed as they included medicated HRT FET cycles only, providing a more homogeneous cohort. HRT FET cycles showed a trend towards a higher PPT in the EC group although still not passing the level of statistical significance (OR 3.06, 95% CI 1.01–9.23; *P *=* *0.05) (Supplementary Fig. S1C).

#### Clinical pregnancy rate

Sixteen studies reported on CPR (8189 in EC group; 16 042 in no EC group). No statistically significant difference in CPR was found between the two groups (OR 1.19; 95% CI 0.99–1.43; *P *=* *0.06) (Supplementary Fig. S2A). No significant difference was found in the sub-group analysis of this data for CPR between ≥5% EC and no EC (OR 1.19; 95% CI 0.84–1.70; *P *=* *0.33), ≥10% EC and no EC (OR 1.11; 95% CI 0.85–1.43; *P *=* *0.45) or ≥15% EC and no EC (OR 1.37; 95% CI 0.69–2.70; *P *=* *0.37).

Definitions of CPR varied significantly across the studies. One study did not define CPR within the manuscript (Bu *et al.*, 2019). A list of the definitions used within the remaining 15 studies is provided in Table 1. To further increase homogeneity, additional sub-analysis of 14 FET cycle-only studies was performed (3968 in EC group; 7181 in no EC group), showing significantly higher CPR in the EC group compared to the no EC group (OR 1.29; 95% CI 1.04–1.61; *P *=* *0.02) (Supplementary Fig. S2B). Further analysis of FET cycles showed that CPR was significantly greater within the EC group on the hormone replacement therapy (HRT)-FET cycle protocol (OR 1.33; 95% CI 1.06–1.67; *P *=* *0.01) but not in those with EC having natural cycle (NC) FETs (OR 1.31; 95% CIs 0.58–2.92; *P *=* *0.52).

To ensure differences in CPR were not being altered due to heterogeneity within this group, sub-analysis was performed on a more homogenous cohort that defined CPR as a viable intrauterine pregnancy with FH seen on ultrasound scan at less than 12 weeks’ gestation, where gestational age was defined. Eight studies were included in this cohort, and CPR was still found to be statistically in favour of EC (OR 1.83; 95% CI 1.15–2.92; *P *=* *0.01) (Supplementary Fig. S2C). Additionally, sub-group analysis of seven FET cycle-only studies with this same definition of CPR (425 in EC group; 690 in no EC group) also showed a statistically significantly CPR in favour of the EC group (OR 2.08; 95% CI 1.28–3.39; *P *=* *0.003) (Supplementary Fig. S2D). Again, this significant improvement in CPR within the EC FET group seemed to be driven by those on HRT-FET cycles (OR 1.73; 95% CI 1.03–2.92; *P *=* *0.04) and not those having NC-FETs (OR 3.35; 95% CI 0.82–13.76; *P *=* *0.09); however, only two studies could be included in the NC-FET meta-analysis, therefore, this finding should be viewed with caution.

Sub-set analysis was performed on four studies which used pre-implantation genetic testing (PGT) for aneuploidy (PGT-A). No significant difference was seen in the CPR between the two groups (OR 0.83; 95% CI 0.63–1.10; *P *=* *0.2). These studies had varying definitions of CPR (Supplementary Fig. S2E).

#### Ongoing pregnancy rate

Eight studies reported OPR as an outcome (1333 In EC group; 5118 in no EC group). This outcome was significantly in favour of EC (OR 1.61; 95% CI 1.09–2.38; *P *=* *0.02) (Supplementary Fig. S3A). When sub-analysing this data based on level of EC, similarly, a significant difference was found in OPR between ≥5% EC and no EC (OR 1.87; 95% CI 1.05–3.34; *P *=* *0.03) and ≥15% EC and no EC (OR 3.52; 95% CI 2.36–5.23; *P *<* *0.00001), both in favour of EC. OPR was found to be in favour of EC when sub-analysing EC at a level of ≥10%, however, this was not found to reach the level of significance (OR 1.50; 95% CI 0.95–2.36; *P *=* *0.08). Sub-set analysis of FET cycles included seven studies and confirmed significantly higher OPR in the EC group (OR 1.79; 95% CI 1.06–3.02; *P *=* *0.03) (Supplementary Fig. S3B), particularly within the HRT-FET cycles (OR 1.87; 95% CI 1.38–2.55; *P *<* *0.0001). Sub-set analysis of euploid embryos only was not possible as only one study that reported OPR used PGT-A (Zilberberg *et al.*, 2020).

#### Miscarriage rate

Twelve papers reported on MR as an outcome. MR was not significantly different between the EC and no EC groups (OR 1.06; 95% CI 0.89–1.25; *P *=* *0.53) (Supplementary Fig. S4A). This result remained when embryos were tested to be euploid prior to transfer (OR 0.63; CI 0.38–1.07; *P *=* *0.09) (Supplementary Fig. S4B). Sub-analysis based on fresh ET (2 studies) and FET cycles (10 studies) confirmed no significant differences in MR (fresh ET cycles: OR 0.78; 95% CI 0.78–1.62; *P *=* *0.54 (Supplementary Fig. S4C); FET cycles: OR 1.04; 95% CI 0.86–1.26; *P *=* *0.7) (Supplementary Fig. S4D).

#### Occurrence of endometrial compaction

**Figure 3.**
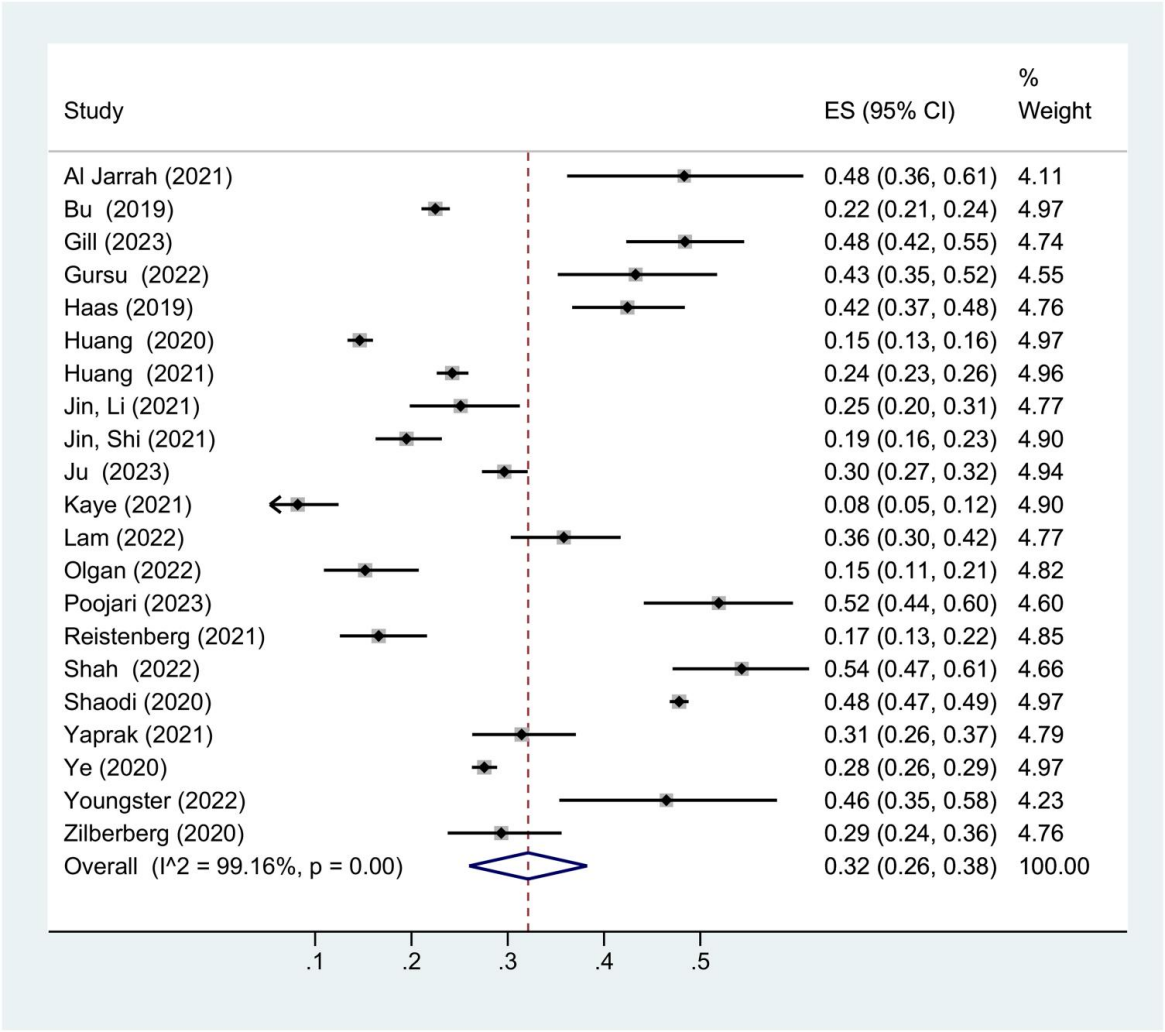
**Forest plot presenting a meta-analysis of the prevalence of EC across all included studies.** EC, endometrial compaction.

Across all 21 studies, the pooled weighted prevalence of EC was 32% (9283 patients, 95% CI 26–38%) versus no EC at 68% (18 574 patients). This suggests that within the general population having IVF/ICSI treatment, EC occurs in a third of patients (Fig. 3).

## Discussion

This meta-analysis includes a total of 27 857 patients undergoing IVF/ICSI treatments across 21 recent research studies and demonstrated that EC is associated with a significant improvement in CPR and OPR but not LBR. Our analysis has also shown the population prevalence of EC to be 32% of patients having IVF/ICSI treatment. However, the positive association of EC for some early pregnancy outcomes did not translate to increased LBR. Therefore, based on current available evidence, stratification of women according to EC cannot yet be justified within clinical practice. Reassuringly, data has shown that EC is not associated with a higher MR or lower PPT or LBR and therefore, is not a negative predictor for favourable pregnancy outcomes. However, our data highlights the assessment of EC as an important and promising area of focus for future studies.

Our findings have shown that LBR is similar between cycles demonstrating EC and cycles without evidence of EC. These results were found to be consistent when sub-analysing for different definitions of EC based on the degree of EMT decrease, with no significant differences between ≥5%, ≥10, or ≥15% EC and no EC. Additionally, no significant difference in LBR was found within PGT-A euploid ET cycles, fresh ET, or frozen (natural or HRT) ET cycles. In keeping with some studies, we also report significantly increased CPR (Jarrah *et al.*, 2021; Yaprak *et al.*, 2021; Youngster *et al.*, 2022; Ju *et al.*, 2023) and OPR with EC (Haas *et al.*, 2019; Zilberberg *et al.*, 2020; Kaye *et al.*, 2021; Youngster *et al.*, 2022). Of the 21 studies we included, only two reported on both OPR and LBR (Huang *et al.*, 2020, 2021) and only four reported on both CPR (defined as a viable intrauterine pregnancy at less than 12 weeks’ gestation) and LBR (Riestenberg *et al.*, 2021; Yaprak *et al.*, 2021; Gursu *et al.*, 2022; Shah *et al.*, 2022), thus, CPR/OPR and LBR results cannot be directly compared due to the large discrepancy in studies included in the meta-analyses and the amount of heterogeneity between the study protocols. However, additional factors to consider that may account for the discrepancy between CPR/OPR and LBR results are obstetric complications that may lead to loss later in pregnancy, and the adequacy of the study power calculation. Due to the natural cumulative loss of pregnancies between PPT through to LBR, a larger sample size is required to achieve a comparable number of live births to clinical/ongoing pregnancies. Within this meta-analysis, we see that a total of 11 149 clinical pregnancies (defined as a viable IUP <12 weeks) were included within the analysis and only 4690 live births, which may also contribute to the discrepancy in the translation of results. The lack of consistency with outcome definitions, cycle protocols, and definitions of EC makes it difficult to draw generalised conclusions from this limited data, and attempting to do so, could potentially lead to spurious results. Only Zilberberg *et al.*, (2020) reported on OPR in PGT-A euploid embryos, precluding a sub-group analysis for this outcome. Haas and Kaye *et al.* included a mix of tested and untested embryos and therefore could not be included in PGT-A sub-group analysis of OPR (Haas *et al.*, 2019; Kaye *et al.*, 2021). Analysis of FET cycles showed significantly better OPR and CPR within the EC versus no EC group, regardless of the definition used for CPR. Sub-group analysis of HRT-FET cycles and NC-FET cycles showed that this significance was likely being driven from the HRT-FET cycles. HRT and natural FET cycles are very different, with natural cycles relying on endogenous production of oestradiol and progesterone (with/without additional luteal supplementation dependent on cycle protocol) and HRT cycles relying on complete artificial endometrial preparation. This difference may be attributed to a difference in serum oestradiol/progesterone levels or because of the comparatively few studies which include NC-FET cycles. Overall, the studies do consistently show that EC is not a negative predictor for pregnancy outcomes. However, according to our findings, it cannot yet be used as a prognostic tool for LBR.

Within this systematic review and meta-analysis, a robust search strategy was implemented using multiple databases and search strategies to ensure the inclusion of all available literature. Where data was unclear or lacking within the manuscripts, authors were contacted directly to supply further clarification. Our protocol was prospectively registered with PROSPERO and performed in accordance with both PRISMA and MOOSE guidelines, ensuring a rigorous study design and high-quality data output. Multiple meta-analyses including subgroup analysis were performed, to ensure the homogenisation of the data analysis as much as possible.

An important limitation of this study is the wide range of heterogeneity identified between the study protocols, including fresh (IVF/ICSI/autologous versus oocyte donor) versus FET cycles (either medicated or natural), different inclusion/exclusion criteria, variation between chosen pregnancy outcomes reported and the definitions used for each pregnancy outcome, study definition of EC, luteal support regimes, method of measuring the EMT (transabdominal (TA) ultrasound versus transvaginal (TV) ultrasound), use of PGT-A, day of ET, and number of embryos transferred. Studies reporting on different pregnancy outcomes as their endpoints, and using different medical protocols, prevented/precluded drawing reliable conclusions from direct comparisons of pooled data. Additionally, 14 out of the 21 studies included were performed as retrospective analyses.

Currently, there is no universally agreed definition for EC. Out of the 21 studies, four defined EC as any decrease in EMT, seven defined EC as > or ≥5% decrease in EMT, four defined EC as ≥10% decrease in EMT, one defined EC as ≥15% decrease in EMT and five did not define EC and therefore it is likely that they used any decrease in EMT as evidence of EC. Due to the lack of definition and studies on the topic, we felt it was important to include all available data on EC within this study, therefore, we used a definition of any decrease in EMT as EC. Whilst we recognise that this increases the heterogeneity in the no EC group, we believe that any significant results obtained would only be further enhanced by greater levels of EC. Additionally, by analysing all available data, this will help plan future studies and develop a definition of EC. Where possible, we sub-analysed the results into different definitions of EC, including ≥5% EC, ≥10% EC, and ≥15% EC, which did not appear to significantly alter the results, and found no change to the OPR or LBR results. When categorising EMT change within the studies, some authors separated the groups into EC and no EC, whilst others separated the no EC group further into unchanged EMT and increased EMT. For the purpose of this study, we compared two groups, EC (defined as any decrease in EMT) and no EC (including those who had no change in EMT and those who had an increase in EMT).

Another important limitation to consider is the method of ultrasound used, the ultrasound technician, and the day of the ultrasound scan. Nine studies used TV ultrasound to measure EMT at all points throughout the cycle. Nine studies used TV ultrasound to measure EMT at the end of the oestrogenic phase and then TA ultrasound to measure EMT around the time of ET. Gursu *et al*. (2022) used TA ultrasound to measure EMT on the first day of progesterone administration and again on the day of ET. Two studies did not specify which method of ultrasound was used. The sensitivity of TV ultrasound is recognised to be superior to TA ultrasound and changes between ultrasound methodology may have introduced intra-observer variability even when performed by the same sonographer, however, this more accurately represents real-world clinical scenarios where it is very common for cycle tracking to be performed under TV ultrasound and TA ultrasound to be used on the day of ET. In some studies, different ultrasound technicians were used for the different scans, increasing the probability of inter-observer variability. Some studies mitigated this by having the EMTs checked by independent practitioners. Due to the well-recognised margin of error that exists when measuring EMT, for future studies, we would advise that having an EC change of at least ≥5% EMT would mitigate this.

Outcome reporting in reproductive medicine studies is a wider and long-standing area of debate (Clarke *et al.*, 2010; Barnhart, 2014; Braakhekke *et al.*, 2014; Gadalla *et al.*, 2018). Different research groups have varying opinions on what pregnancy outcome is the most meaningful within a trial setting (Clarke *et al.*, 2010; Barnhart, 2014; Braakhekke *et al.*, 2014; Gadalla *et al.*, 2018). In 2003, ESHRE recommended that the outcome measure for ART and non-ART should be ‘singleton live birth rate’ (Land and Evers, 2003). Some argue that OPR serves as a better primary outcome, as this eliminates confounding factors such as second-trimester loss, stillbirth, multiple pregnancies, and the number of embryos transferred, hence why occasionally some studies report on the IR as an outcome (Dickey *et al.*, 2004). Additional arguments against LBR include the need for a larger sample size, as LBR is lower than other pregnancy outcomes; the need for longer trials, increasing the risk of patients being lost to follow-up, increasing costs and delaying results and difficulties due to fragmentation between gynaecological and obstetric care (Braakhekke *et al.*, 2014). However, due to the inconsistencies in definitions, even other pregnancy outcomes such as biochemical pregnancy rate, CPR or OPR may fall into different categories relevant to different studies, depending on the specific gestational cut-off used. Therefore, until outcomes are reported homogeneously, all pooled data should be viewed with an element of caution (Barnhart, 2014).

Having a live birth is the reason why sub-fertile patients seek ART, and therefore LBR should be the outcome of focus and should be reported on in every trial performed within reproductive medicine. Standardising outcome definitions is essential to ensure that we are producing reliable, meaningful, and translatable data for use in clinical practice and, thus, ultimately, we can provide better care to our patients.

## Conclusions

EC may significantly improve CPR and OPR, although this is not yet seen to translate to LBR. This may be masked due to the heterogeneity between the studies or due to a lack of currently available data. However, for patients, LBR is understandably the most important pregnancy outcome, and therefore currently, EC should not be used to stratify patients in clinical practice. Nevertheless, EC may hold promise for the future as a non-invasive predictor of positive pregnancy outcomes and, therefore, should be a focus for a future clinical trial, including LBR as the primary outcome. We recommend a large prospective multicentre clinical trial with a robust protocol, ensuring minimal cycle variability, clear definitions for EC and pregnancy outcomes, and TV sonography to be performed throughout by highly trained ultrasound technicians and reviewed by independent technicians according to pre-determined criteria. If shown to be of benefit, EC has many advantages over currently available invasive and costly methods of testing for endometrial receptivity, which have little robust evidence to support them. Additionally, in patients with RIF, where many cases remain unexplained, EC could act as a useful tool to aid appropriate counselling and management of patients for a successful pregnancy.

## Supplementary Material

hoae040_Supplementary_Data

## Data Availability

The data underlying this article can be shared on reasonable request to the corresponding author.
