# Supplementary figures and images for: Endometrial compaction to predict pregnancy outcomes in patients undergoing assisted reproductive technologies: a systematic review and meta-analysis
Source: Hum Reprod Open. 2024 Jun 20;2024(3):hoae040. doi: 10.1093/hropen/hoae040 (PMC11239225; doi:10.1093/hropen/hoae040)

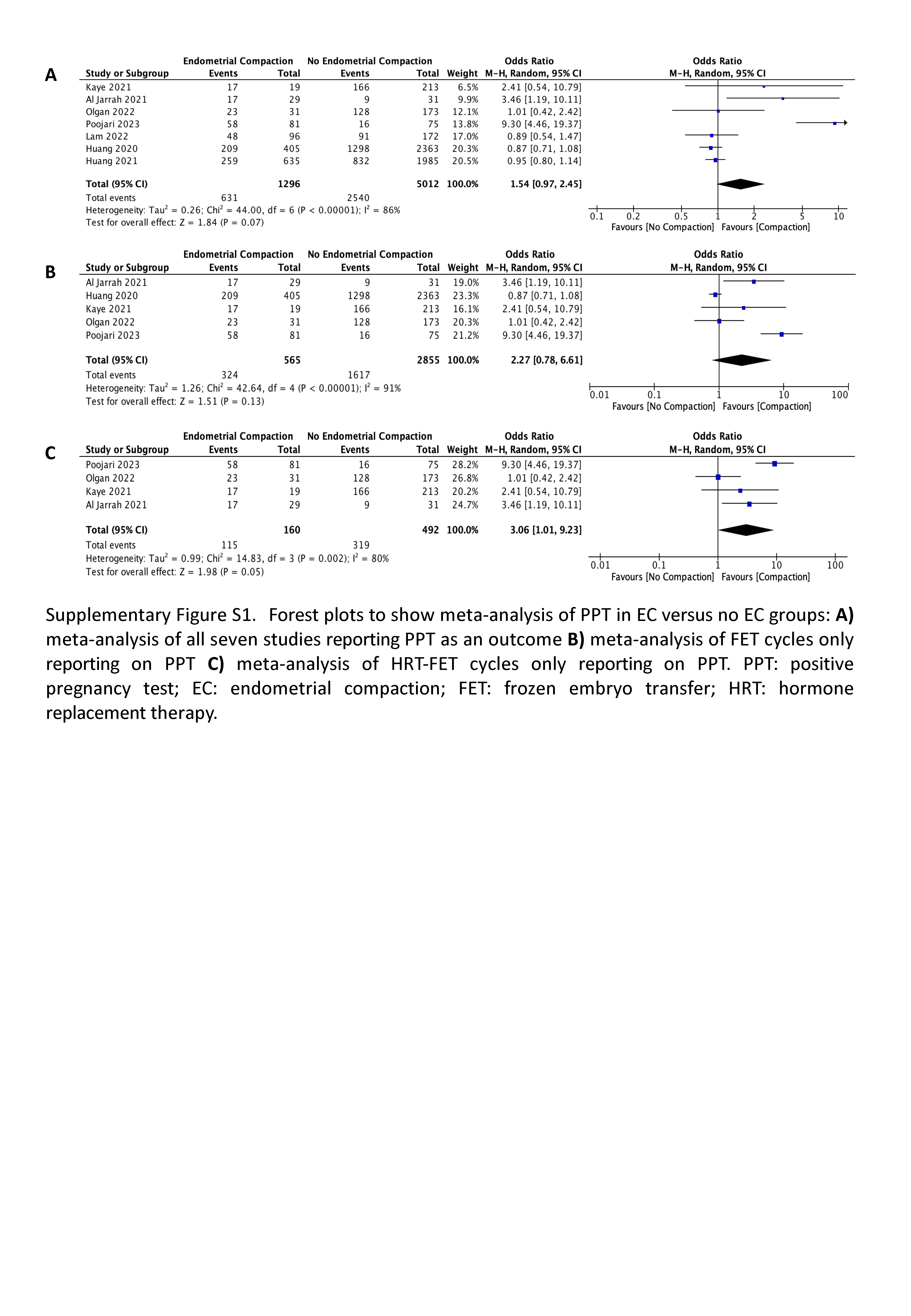

Supplement: hoae040_Supplementary_Data [file hoae040_supplementary_data.zip › R2_SuppFig_S1_HRO_PPT.tiff]

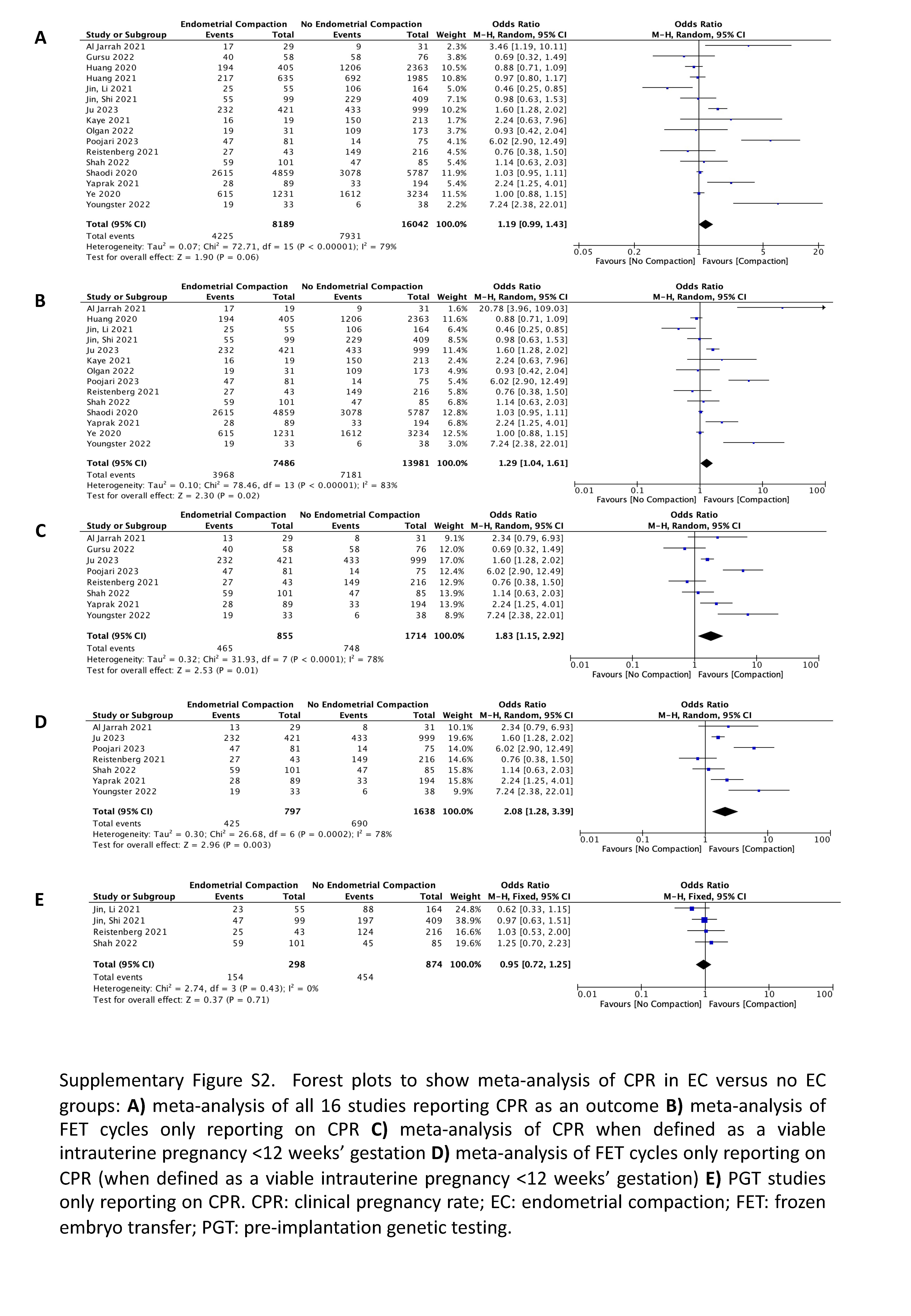

Supplement: hoae040_Supplementary_Data [file hoae040_supplementary_data.zip › R4_SuppFig_S2_Rev.tiff]

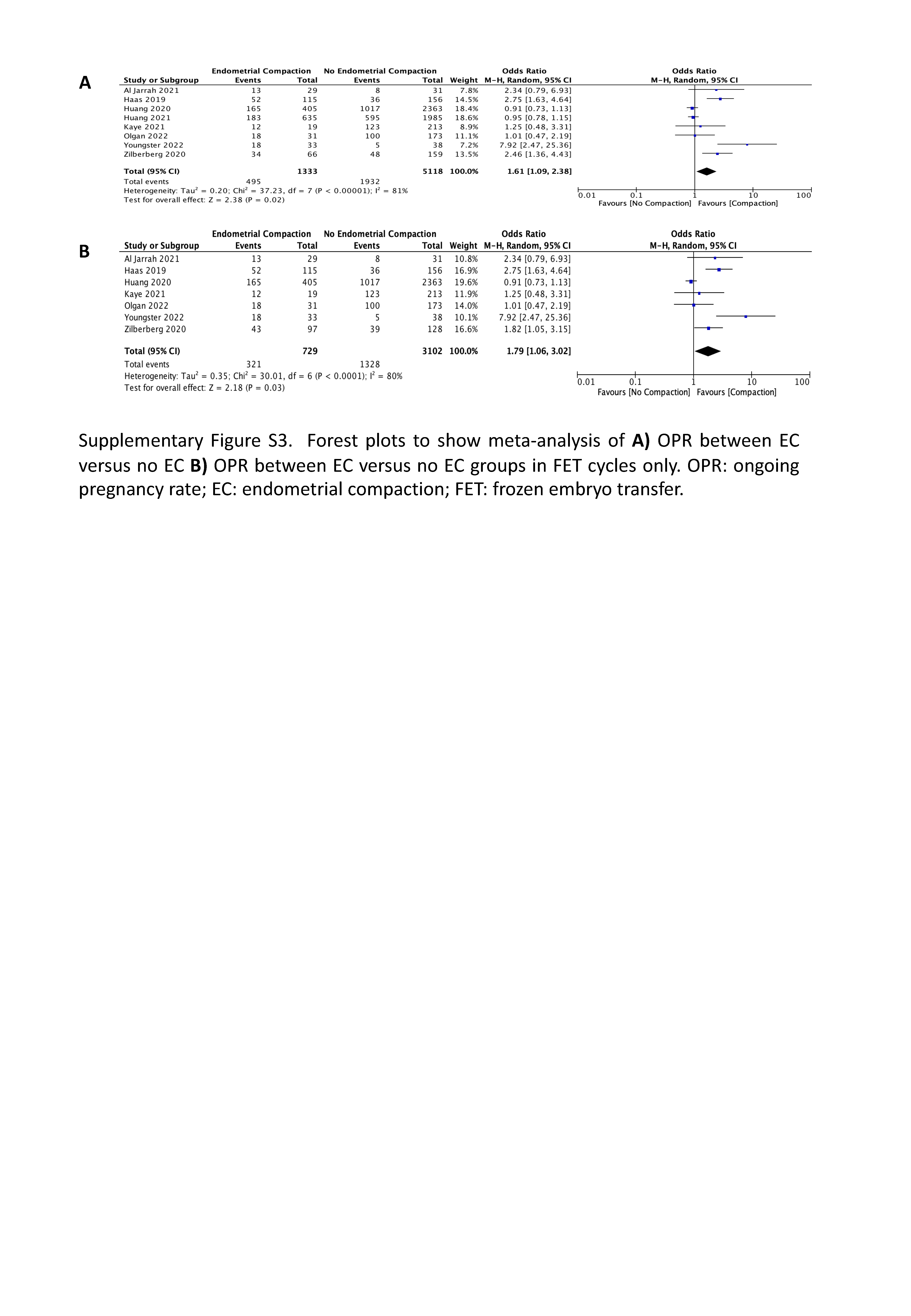

Supplement: hoae040_Supplementary_Data [file hoae040_supplementary_data.zip › R4_SuppFig_S3_Rev.tiff]

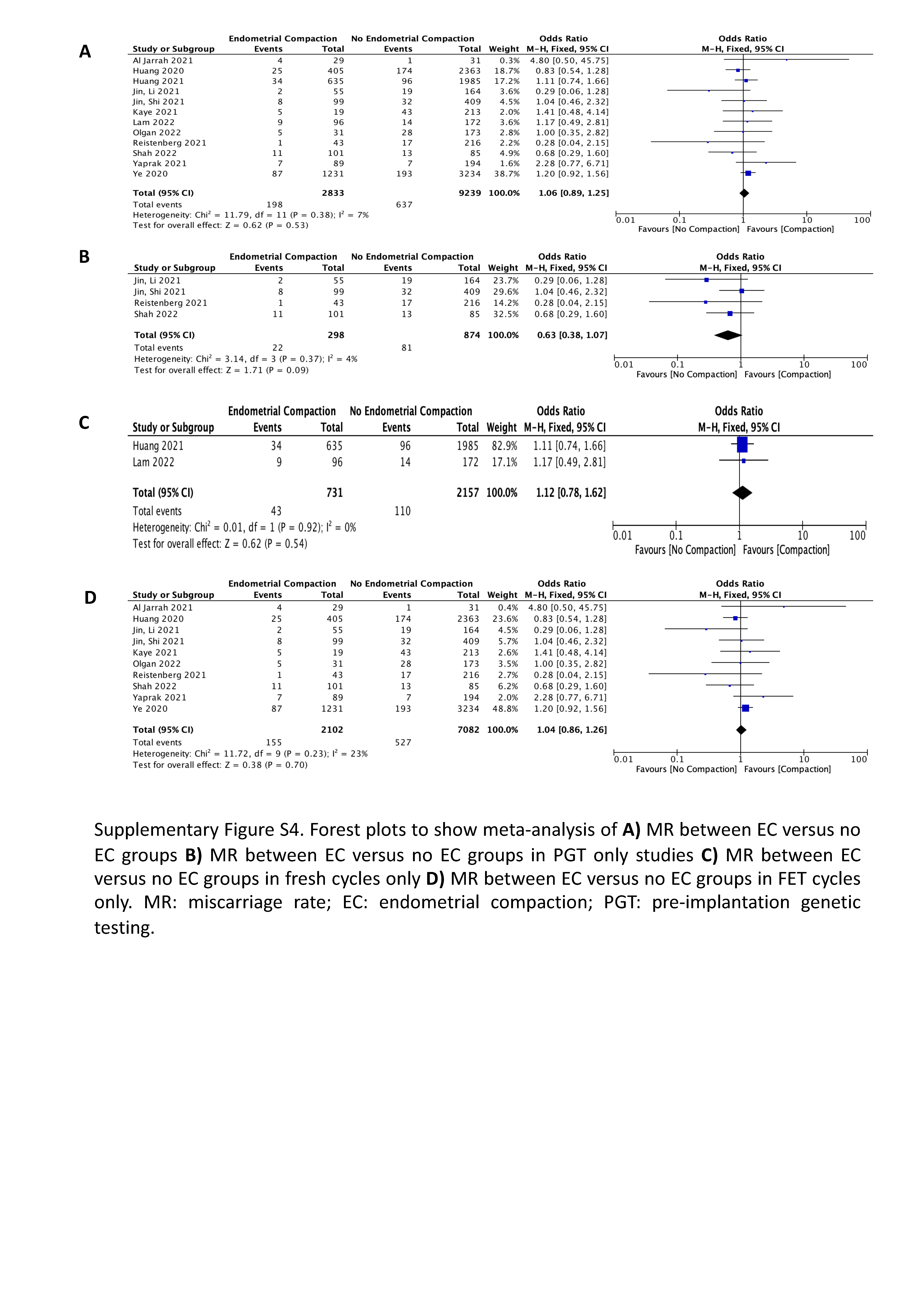

Supplement: hoae040_Supplementary_Data [file hoae040_supplementary_data.zip › R4_SuppFig_S4_Rev.tiff]
